# Comparison of dimethyl fumarate and interferon outcomes in an MS cohort

**DOI:** 10.1186/s12883-022-02761-8

**Published:** 2022-07-11

**Authors:** Neda Sattarnezhad, Brian C. Healy, Moogeh Baharnoori, Camilo Diaz-Cruz, James Stankiewicz, Howard L. Weiner, Tanuja Chitnis

**Affiliations:** 1 Harvard Medical School, Boston, Massachusetts 02115 USA; 2 Brigham Multiple Sclerosis Center, Brigham and Women’s Hospital, Boston, Massachusetts 02115 USA; 3 Biostatistics Center, Massachusetts General Hospital, Boston, Massachusetts USA

**Keywords:** Multiple sclerosis, Dimethyl fumarate, Interferon, Disease activity, Effectiveness, Cohort

## Abstract

**Background:**

To compare the effectiveness of dimethyl fumarate (DMF) with subcutaneous interferon beta-1a (IFNβ-1a) in controlling disease activity in patients with relapsing–remitting Multiple Sclerosis (MS).

**Methods:**

Clinical and imaging data from patients treated with either IFNβ-1a or DMF for at least one year were reviewed. The proportion of patients with at least one clinical relapse within 3–15 months after treatment onset, the proportion of patients with new T2 or gadolinium-enhancing lesions, and the proportion of subjects who achieved no evidence of disease activity (NEDA) status were assessed.

**Results:**

Three hundred sixteen (98 on IFNβ-1a, 218 on DMF) subjects were included. Baseline demographics were comparable between groups except for age, disease duration, and the number of previous treatments being higher and relapse rate in the prior year being lower in the DMF-treated group. The proportion of patients having a clinical relapse (24.5% vs. 9.6%; OR = 3.04; *P* < 0.001) or a new MRI lesion (28.6% vs. 8.7%; OR = 4.19, *P* < 0.001) at 15 months were higher on IFNβ-1a. 79.9% of the patients achieved NEDA status at 15 months on DMF (vs. 51.1% for IFNβ-1a; OR = 0.26, *P* < 0.001). Further adjustment for demographics, disease characteristics, treatment and relapse history, and subgroup analyses confirmed these findings.

**Conclusion:**

DMF was associated with less clinical and radiological disease activity compared to IFNβ-1a.

**Supplementary Information:**

The online version contains supplementary material available at 10.1186/s12883-022-02761-8.

## Background

Multiple sclerosis (MS) is the most common demyelinating disease of the nervous system and a major cause of lifelong disability in the young adult population [1]. The first generation of disease-modifying therapies (DMT) for MS was approved in the 1990s to modify the course of the disease. Interferonβ (IFNβ-1a) and glatiramer acetate, as the first approved treatments, started a new era in the management of MS [2]; however, to date, limited effectiveness and route of administration have been the main reasons for poor adherence to these medications. These factors raise the need for more effective treatments with a more convenient method of usage [3–9].

Dimethyl fumarate (DMF) is an oral treatment approved by the FDA to treat relapsing–remitting multiple sclerosis (RRMS). Induction of nuclear factor-erythroid 2-related factor 2 (Nrf2) antioxidant pathway and shifting cell differentiation toward Th2 immune response are the main accepted mechanisms of action for dimethyl fumarate (DMF) which suggest both anti-inflammatory and neuroprotective roles of the drug [10–12]. Phase 2 and 3 placebo-controlled clinical trials reported DMF to decrease annualized relapse rate (ARR) in treated patients by 50% and control subclinical disease activity more effectively [13–15]. Glatiramer acetate (GA) was used as a reference comparator in phase 3 (CONFIRM) trial. However, no published studies compare the effectiveness of DMF to IFNβ-1a, a widely studied first-generation MS treatment with a known safety profile.

This study aimed to compare the effectiveness of DMF to IFNβ-1a in controlling disease activity and achieving no evidence of disease activity (NEDA) status in patients with RRMS.

## Methods

### Subjects

For this retrospective cohort study, patients were selected from an ongoing longitudinal study at our institution entitled, Comprehensive Longitudinal Investigation of Multiple Sclerosis at the Brigham and Women’s Hospital (CLIMB) [16]. The CLIMB study started recruiting subjects in 2000 and is approved by the institutional review board (IRB) of Partners Health System, and the current study was approved by IRB as an amendment to the CLIMB study protocol. The patients have been enrolled after providing informed written consent. Patients undergo a semiannual clinical evaluation including expanded disability status scale –EDSS-measurement [17], an annual brain and a biannual spine MRI using a standardized protocol. All patient data are recorded in an Oracle-based database.

### Data collection

The inclusion criteria for the current study were: i) age of 18–55 years at disease onset, ii) treatment with subcutaneous (SC) interferon-b1a (IFNß-1a; Rebif; Merck-Serono) or oral dimethyl fumarate (DMF; Tecfidera; Biogen Idec) for at least 12 months, iii) relapsing–remitting course of the disease, based on McDonald criteria 2010,18 iv) EDSS < 6 on treatment onset, and (v) treatment initiation after 1/1/2008. Progressive disease course or concomitant treatment with other DMTs resulted in exclusion from the study. Clinical and imaging data were retrieved for included subjects from our validated Oracle-based database, based on the patient medical records.

### Endpoints

Our primary endpoint was the occurrence of at least one relapse between 3–15 months after being started on IFNβ-1a or DMF. A relapse was defined as new or recurrent patient-reported or objectively recorded neurologic abnormalities typical for a demyelinating event lasting for more than 24 h in the absence of a recent infection or fever [18].

The secondary endpoints were the proportion of subjects who had a new gadolinium-enhancing or T2-hyperintense lesion on a follow up brain MRI within 3–15 months after the medication start date, the proportion of patients with sustained disease progression, and the proportion of subjects who achieved the composite endpoint of no evidence of disease activity (NEDA) status (no relapse, new MRI lesion or sustained disease progression) at 15 months. Baseline MRI brains were obtained within the first 6 months after treatment (IFN vs. DMF) initiation. Sustained disease progression was defined as an increase in EDSS that lasted for at least 180 days. For subjects with a pretreatment EDSS of 0, the increase was 1.5 units; for subjects with a pretreatment EDSS of 1–5, the increase was 1 unit; for subjects with a pretreatment EDSS of 5.5 or 6, the increase was 0.5 units.

### Statistical analysis

Baseline demographics, disease duration at treatment start, EDSS at treatment onset, the relapse rate in the year prior to treatment, and the number of previous courses on other treatments were compared between groups using independent samples t-test, chi-square test, and Mann–Whitney U test as appropriate. Our initial analysis compared the two treatment groups in each outcome using a univariate logistic regression model for dichotomous outcomes. Given the significant differences between the groups at baseline, we adjusted for confounders using three commonly used approaches to compare treatment groups with observational data. The confounders included in our models were age, gender, disease duration, EDSS, number of relapses in the year prior to treatment, previous treatments with IFN, previous treatment with GA, and previous treatment with any other DMTs. Our first approach used a multivariable logistic regression analysis to estimate the adjusted odds ratio comparing the two treatment groups controlling for the other factors. Second, we fit a propensity score model using the same set of confounders, and we adjusted for the propensity score in our logistic regression model. Third, we used the inverse probability of treatment weighting to estimate the average treatment effect. Each subject was weighted by the inverse of the probability of the treatment that they received. To assess the balance between the treatment groups for the inverse probability weighted model, we calculated the demographic characteristics of the groups in the weighted sample and compared the balance between the weighted groups.

Given the potential for residual confounding even after regression adjustment, controlling for the propensity score or inverse probability weighting, several sensitivity analyses were performed to assess whether our conclusions were robust. First, a large proportion of subjects switched from another treatment to DMF based on patient preference, potentially due to a preferred mode of delivery rather than the lack of effectiveness of the previous treatment. Thus, we refit all our models only in subjects who switched from previous treatment for a reason other than patient preference. Second, we refit our analyses only in subjects who switched from previous DMT due to disease activity. For each of the earlier analyses, the reason for treatment switching was derived by one rater (NS) from the physician note and added to our database. Third, to further focus attention on subjects who likely switched due to disease activity, we refit the model only in subjects who had at least one clinical relapse within the 12 months prior to treatment start. Fourth, to remove the potential differences in older onset MS patients and to make our sample like previous randomized clinical trials, we refit the model including only subjects who were between 18–55 years at treatment (IFN vs DMF) onset and including only subjects who were between 18–55 years at treatment (IFN vs DMF) onset who also had a relapse within the previous year. A two-sided alpha level of 0.05 was used to assess statistical significance for all the analyses. All statistical analyses were completed in the statistical package R (www.r-project.org).

## Results

### Demographics and disease features

A total of 316 patients met the inclusion criteria and contributed to the analysis. Ninety-eight and 218 subjects were treated with IFNβ-1a and DMF, respectively. Baseline demographics and disease features were shown in Table 1. The gender distribution was similar between groups, and most of the subjects were female. Patients treated with IFNβ-1a were younger, had lower disease duration and higher relapse rate in the year prior to the treatment start date (*P* < 0.001). The baseline EDSS was comparable between groups (*P* = 0.13).

**Table 1:** Demographic characteristics of study groups

	IFN-b 1a	DMF	*P*-value
N	98	218	
Race (% White)	86 (87.8)	195 (89.4)	0.80
Female (%)	66 (67.4)	160 (73.4)	0.44
Age (years, mean ± SD)	38.33 ± 10.64	45.91 ± 10.44	< 0.001
Disease duration (years, mean ± SD)	6.76 ± 7.67	11.94 ± 8.31	< 0.001
EDSS at treatment initiation (mean ± SD)	1.43 ± 1.00	1.67 ± 1.40	0.13
Treatment-naïve patients (%)	34 (34.7)	29 (13.3)	< 0.001
Relapses in year prior to treatment (mean ± SD)	1.05 ± 0.95	0.32 ± 0.57	< 0.001
Number of previous treatments (mean ± SD)	0.99 ± 1.09	1.94 ± 1.76	< 0.001
Previous course of IFN (N (%))	39 (39.8)	98 (45.0)	0.46
Previous course of GA (N (%))	38 (38.8)	121 (55.5)	0.01
Previous course of other treatments (N (%))	8 (8.2)	57 (26.2)	< 0.001
Reason for stopping previous treatments (N (%))			< 0.001
Disease activity	38 (60.3)	49 (27.1)	
Intolerance/ allergy	3 (4.8)	23 (12.7)	
No information	2 (3.2)	10 (5.5)	
Other	12 (19.0)	32 (17.7)	
Patient preference	3 (4.8)	51 (28.2)	
Side effect	5 (7.9)	16 (8.8)	

*Legend*: *IFN-b 1a* Interferon beta-1a, *DMF* Dimethyl Fumarate, *GA* Glatiramer Acetate, *EDSS* Expanded Disability Status Scale, *SD* Standard Deviation

In terms of clinical relapses, 24 patients in the IFNβ-1a group (24.5%) had at least one clinical relapse within the study period compared to 21 patients (9.6%) in the DMF group (OR = 3.04; *p* < 0.001), as shown in Table 2.

**Table 2:** Comparison of treatment groups across clinical and radiologic outcomes in the interval between 3 and 15 months after treatment initiation

	IFNb-1a	DMF	OR^a^ (95% CI)
Number (%) of subjects with a relapse	24 (24.5)	21 (9.6)	3.04 (1.60, 5.79)
Number (%) of subjects with a new lesion on brain MRI	28 (28.6)	19 (8.7)	4.19 (2.20, 7.97)
Number (%) of subjects with a new GD + lesion on brain MRI	12 (12.2)	12 (5.5)	2.40 (1.04, 5.54)
Number (%) of subjects with new T2 lesion on brain MRI	26 (26.5)	17 (7.8)	4.27 (2.19, 8.33)
Number (%) of subjects with sustained disease progression	8 (8.9)	12 (5.6)	1.64 (0.65, 4.17)
Number (%) of subjects with no relapse, new MRI lesion or sustained progression (NEDA)	46 (51.1)	171 (79.9)	0.26 (0.15, 0.45)
			RR (95% CI)^b^
Annualized relapse rate	0.29	0.11	2.49 (1.40, 4.44)

*Legend*: *IFN-b 1a* Interferon beta-1a, *DMF* Dimethyl Fumarate, *OR* Odds Ratio, *RR* Rate ratio, *CI* Confidence Interval, *GD* + Gadolinium-enhancing, *NEDA* No Evidence of Disease Activity. ^a^OR > 1 indicates higher probability of having an event on IFNb-1a compared to DMF. ^b^RR and associated 95% CI were calculated using Poisson regression with overdispersion

Three hundred twenty-two MRI scans (184, 1.5 T and 138, 3 T) were monitored for evidence of radiological disease activity according to the reports from a neuroradiologist or MS specialist. Patients treated with IFNβ-1a also had a higher risk of developing a new MRI lesion (OR = 4.19; *p* < 0.001), and this was driven primarily by new T2 lesions rather than GD + lesions (Table 2). The proportion of patients who had sustained disease progression was similar in both groups (OR = 1.64; *P* = 0.30); however, a lower proportion of patients achieved NEDA status in the IFNβ-1a group (OR = 0.26; *p* < 0.001; Table 2).

Given the differences between the groups at the time of treatment choice, we used three approaches to adjust for potential confounders. When we adjusted for the confounders using multivariable logistic regression (Table 3), the IFNβ-1a treated group had a higher risk of having a relapse (OR = 3.43; *P* = 0.001) and a reduced chance of maintaining NEDA (OR = 0.27; *p* < 0.001). In addition to the multivariable logistic regression model, we also used logistic regression to estimate the propensity score, and the estimated propensity score model is presented in Supplementary Table 1. When we controlled for the propensity score, similar associations were observed as in the previous analysis even though the magnitude of the difference between groups was reduced (Table 3). Finally, when we used inverse probability weighting as the final approach to handle confounding, we compared the balance of the treatment groups in the weighted sample, and a reasonable balance was achieved (Supplementary Table 2). When the treatment groups were compared in the weighted sample, we found smaller differences between the groups than in the previous approaches to account for confounding. Still, the overall conclusions were consistent (Table 3). Overall, adjustment for confounding still showed improved disease course for subjects in the DMF group.

**Table 3:** Comparison of treatment groups across clinical and radiologic outcomes in the interval between 3 and 15 months after treatment initiation using approaches to address confounding

Outcome	Regression adjustment for all confounding factors OR (95%CI)	Regression adjustment for propensity score OR (95%CI)	Inverse probability weighting OR (95%CI)
Clinical relapse(s)	3.43 (1.55, 7.60)	2.84 (1.33, 6.06)	2.34 (0.86, 6.72)
New lesion on brain MRI	4.40 (2.04, 9.50)	4.30 (2.02, 9.14)	3.77 (1.67, 8.99)
New GD + lesion on brain MRI	2.32 (0.87, 6.21)	2.26 (0.84, 6.08)	2.03 (0.67, 5.91)
New T2 lesion on brain MRI	4.89 (2.21, 10.85)	4.98 (2.27, 10.91)	4.11 (1.79, 10.04)
Sustained disease progression	1.32 (0.39, 4.42)	1.09 (0.36, 3.32)	0.92 (0.28, 2.44)
No relapse, new MRI lesion or sustained progression (NEDA)	0.27 (0.14, 0.50)	0.30 (0.16, 0.56)	0.35 (0.15, 0.75)

*Legend*: *OR* Odds Ratio, *CI* Confidence Interval, *Gd* + Gadolinium-enhancing, *NEDA* No Evidence of Disease Activity. Estimated OR and 95% CI provided for each of the outcomes for each of the three approaches. OR > 1 indicates higher probability of having an event on IFNb-1a compared to DMF

### Sensitivity analyses

To ensure that our conclusions were robust, several sensitivity analyses were performed. In the subgroup of patients who did not stop previous treatment due to personal preference (n = 262), the estimated difference between the treatments (IFN vs DMF) was similar as in the primary analyses (Supplementary Table 3). In the subgroup of patients who stopped their previous treatment due to disease activity (n = 87), the estimated difference between the treatments was like the primary analysis even though the confidence intervals were wider due to the considerable reduction in sample size (Supplementary Table 4). When we focused on the subjects who reported relapse in the previous year (n = 128), the estimated difference between the treatments was similar (Supplementary Table 5). Finally, when we analyzed subjects between 18–55 years old (n = 261) and subjects between 18–55 years old who had a relapse in the prior year (n = 115), the estimated differences were similar (Supplementary Tables 6 and 7).

## Discussion

We compared DMF versus IFNβ-1a in patients with relapsing–remitting multiple sclerosis. In our sample, 9.6% of the patients treated with DMF had at least one relapse within 3–15 months after treatment onset compared to 24.5% in the IFNβ-1a group. The IFNβ-1a treated subjects had a significantly higher risk of relapse, and the difference stayed significant after adjustment for baseline demographics, disease characteristics, and treatment history. Restricting the comparison only to the subjects who were treatment-naïve before DMF or IFNβ-1a start date showed similar results (data not shown). The estimated difference between the treatments based on our sample is much more significant than reported in the network meta-analysis, so it must be interpreted cautiously [19].

The major difference between our sample and the clinical trials of both treatments was the very low relapse rate in subjects treated with DMF observed in our study. Phase III clinical trials of DMF have reported 24–29% of patients have at least one clinical event after 2 years on DMF [14, 15]. Clinical trial patients had annualized relapse rate (ARR) of 0.14–0.22 on DMF [13–15, 20], while the ARR in our group of CLIMB subjects treated with DMF was 0.1. There are several possible explanations for the different rates of disease activity in our study compared to clinical trials. First, while clinical trials included all events occurring from the first day after medication intake, we initiated monitoring for disease activity 3 months after treatment initiation, when these medications had reached their maximum biological effects. This allowed us to detect breakthrough activities, most likely attributable to suboptimal disease control of IFN or DMF. Second, a longer follow-up period in trials (2-years versus 1 year) may result in the detection of more clinical events. Third, our study involves subjects from a single center, which may reduce the heterogeneity of our study population. Fourth, DMF-treated patients in our MS cohort, on average, were older and had higher disease duration compared to most clinical trials, which may decrease the probability of having a relapse compared to the younger population [21, 22]. Finally, it is possible that the subjects who were placed on DMF were considered healthier by their physician so that the relapse rate in this set of subjects would be lower than in the trials.

In a real-world propensity-matched comparative analysis, DMF was similar to fingolimod in controlling inflammatory disease activity and disability progression [23]. Considering superiority of fingolimod to interferon beta-1a [24], these results are in agreement with our findings on better control of inflammatory disease activity on DMF compared to the interferons. Findings from the Italian MS register is also suggestive of lower relapse rate on oral DMTs (DMF and teriflunomide) compared to the injectables (copaxone and interferons) [25].

The reported disease activity in IFNβ-1a (SC) clinical trials has changed since the early trials. These changes parallel the overall changes in the natural history of the disease [26], from ARR of 0.54 [22, 27] and relapse-free rate of 45–62% at 1-year [9, 28] to ARR of 0.35–0.4 [29–33] and relapse-free rate of 60–62% at 2-year, 51–57% at 3-year [29, 31, 32], and 46% in 5-year [30]. Calculated ARR for our patients was 0.31 on IFNβ-1a, and 75% stayed relapse-free from 3–15 months after treatment start. These estimates are like the recent clinical trials.

An important limitation of our treatment comparison is that almost all the subjects placed on IFNβ-1a were put on treatment before the approval of DMF. Thus, few of the subjects in our analysis had the opportunity to choose between these two treatments at the time of the treatment decision. We attempted to limit the differences between the treatment groups by including only subjects starting IFNβ-1a after 1/1/2008, but we acknowledge that some relevant additional confounding factors may remain. To minimize the effects of dissimilarities in treatment arms, several subgroup analyses and approaches to adjust for possible confounders were employed, and all of these analyses confirmed the main findings. It is also important to note that the increased number of available DMTs in recent years may have lowered the threshold used by clinicians to define treatment failure. Since DMF was the most recent approved oral medication for MS, neurologists had more options when this medication was released into the market. Thus, it is possible that the patients who stayed on DMF for at least 12 months were less likely to have experienced a relapse or MRI activity. Therefore, our inclusion criterion of a minimum of 12 months on treatment may have led to the selection of patients with better treatment response and exclusion of poor-treatment responders earlier than meeting this criterion. To assess this potential bias, we compared our results to the patients who had a relapse within 3 to 15 months after the treatment start date (regardless of treatment duration) in our larger CLIMB cohort. The numbers for the whole cohort (23% on IFNβ-1a and 8% on DMF) were like the findings of this study.

A strength of our study was the assessment of NEDA status in both treatment groups. The success rate in inducing no evidence of disease activity (NEDA) status consists of no clinical relapses, no subclinical MRI activity (no new/enlarging T2 or Gadolinium-enhancing lesions), and no disease progression, is becoming the ideal measure to define the effectiveness of DMTs [34]. Clinical relapses in combination with local inflammatory activity on MRI in the first years of treatment are the main predictors of disease progression and disability later in the disease course [35–37]. Studies report about 28% and 27% of the patients having NEDA at 2-years on DMF and IFNβ-1a, respectively [37, 38]. In our study, the analysis of NEDA showed a similar pattern as the other analyses, with a higher number of patients being disease-free at 15 months (80% on DMF and 51% on IFNβ-1a). In addition to showing the same effect as the other metrics, the increased number of events observed when combining the two measures (clinical and radiologic findings) leads to an increase in power. This result could justify this outcome in future short-term trials in MS.

Although our results suggest that RRMS patients treated with DMF have a higher success rate in achieving NEDA status compared to IFNβ-1a, lack of clinical trials comparing the efficacy and effectiveness of DMF versus IFNβ-1a in relapsing–remitting multiple sclerosis did not allow us to compare our results with the so-called gold standard of clinical research. Therefore, clinical trials enabling a head-to-head comparison of DMF and IFNβ-1a are necessary to elucidate our findings further.

## Conclusions

In our study, IFNβ-1a treated patients had a significantly higher number of relapses after adjustment for baseline demographics, disease characteristics, and treatment history. Similar to the clinical endpoint, the number of new MRI lesions was significantly increased in patients treated with IFNβ-1a, which was mainly driven by new T2 lesions. Also, a higher number of DMF treated patients remained disease-free at 15 months on treatment. Sensitivity analyses confirmed the primary findings. Clinical trials are needed to confirm the results of this study.

## Supplementary Information

**Additional file 1:** **Supplementary table 1.** Logistic regression model for propensity score.

**Additional file 2:** **Supplementary table 2.** Demographic characteristics of study groups in inverse probability weighted sample.

**Additional file 3:** **Supplementary table 3.** Comparison of treatment groups among subjects who did not change from previous treatment based on patient preference.

**Additional file 4:** **Supplementary table 4.** Comparison of treatment groups among subjects who changed from previous treatment based on disease activity.

**Additional file 5:** **Supplementary table 5.** Comparison of treatment groups among subjects who had a relapse in the previous year.

**Additional file 6:** **Supplementary table 6.** Comparison of treatment groups among subjects who were between 18 and 55 at the time of treatment initiation.

**Additional file 7:** **Supplementary table 7.** Comparison of treatment groups among subjects who were between 18 and 55 at the time of treatment initiation and had a relapse in the previous year.

## Data Availability

The authors affirm that this manuscript is an honest, accurate and transparent account of the study being reported and no important aspects of the study have been omitted. The raw data will be shared with the qualified researchers upon reasonable request after publication of this manuscript.

## Abbreviations

MS: Multiple Sclerosis
RRMS: Relapsing–Remitting Multiple Sclerosis
DMF: Dimethyl Fumarate
IFNβ-1a: Interferon Beta-1a
SC: Subcutaneous
GA: Glatiramer Acetate
NEDA: No Evidence of Disease Activity
ARR: Annualized Relapse Rate
DMT: Disease Modifying Treatment
EDSS: Expanded Disability Status Scale
CLIMB study: Comprehensive Longitudinal Investigation of Multiple Sclerosis at the Brigham and Women’s Hospital study
