# Supplementary material for: Comparison of dimethyl fumarate and interferon outcomes in an MS cohort
Source: BMC Neurol. 2022 Jul 11;22:252. doi: 10.1186/s12883-022-02761-8 (PMC9277810; doi:10.1186/s12883-022-02761-8)
Supplement: Supplementary file 1 — Additional file 1: Supplementary table 1. Logistic regression model for propensity score. [file 12883_2022_2761_MOESM1_ESM.docx]

Supplementary table 1: Logistic regression model for propensity score

|  | Logistic regression coefficient | p-value |
| --- | --- | --- |
| Age | -0.05 | 0.005 |
| Male | 0.30 | 0.54 |
| Disease duration | -0.02 | 0.44 |
| Number of attacks in previous year | 1.21 | <0.01 |
| EDSS | -0.01 | 0.92 |
| Previous treatment with IFN | 0.55 | 0.09 |
| Previous treatment with GA | -0.22 | 0.46 |
| Previous treatment with other treatments | -1.19 | 0.01 |

Legend: EDSS: Expanded Disability Status Scale; IFN: Interferon; GA: Glatiramer Acetate. The estimated area under the ROC curve for this model was 0.811.
