# Supplementary material for: Comparison of dimethyl fumarate and interferon outcomes in an MS cohort
Source: BMC Neurol. 2022 Jul 11;22:252. doi: 10.1186/s12883-022-02761-8 (PMC9277810; doi:10.1186/s12883-022-02761-8)
Supplement: Supplementary file 2 — Additional file 2: Supplementary table 2. Demographic characteristics of study groups in inverse probability weighted sample. [file 12883_2022_2761_MOESM2_ESM.docx]

Supplementary table 2: Demographic characteristics of study groups in inverse probability weighted sample

|  | IFN-b 1a | DMF | p-value |
| --- | --- | --- | --- |
| Female (%) | 0.74 | 0.73 | 0.92 |
| Age (years, mean) | 43.94 | 44.34 | 0.76 |
| Disease duration (years, mean) | 10.50 | 10.78 | 0.79 |
| EDSS at treatment initiation (mean) | 1.61 | 1.69 | 0.63 |
| Relapses in year prior to treatment (mean ± SD) | 0.57 | 0.62 | 0.61 |
| Previous course of IFN (N (%)) | 0.44 | 0.45 | 0.86 |
| Previous course of GA (N (%)) | 0.50 | 0.54 | 0.53 |
| Previous course of other treatments (N (%)) | 0.21 | 0.22 | 0.81 |

Legend: IFN: Interferon; DMF: Dimethyl Fumarate; GA: Glatiramer Acetate; EDSS: Expanded Disability Status Scale; SD: Standard Deviation.
