# Supplementary material for: Comparison of dimethyl fumarate and interferon outcomes in an MS cohort
Source: BMC Neurol. 2022 Jul 11;22:252. doi: 10.1186/s12883-022-02761-8 (PMC9277810; doi:10.1186/s12883-022-02761-8)
Supplement: Supplementary file 3 — Additional file 3: Supplementary table 3. Comparison of treatment groups among subjects who did not change from previous treatment based on patient preference. [file 12883_2022_2761_MOESM3_ESM.docx]

Supplementary table 3: Comparison of treatment groups among subjects who did not change from previous treatment based on patient preference

| Outcome | Unadjusted  OR (95%CI) | Regression adjustment for all confounding factors  OR (95%CI) | Regression adjustment for propensity score  OR (95%CI) | Inverse probability weighting  OR (95%CI) |
| --- | --- | --- | --- | --- |
| Clinical relapse(s) | 3.01 (1.50, 6.05) | 2.93 (1.26, 6.83) | 2.47 (1.10, 5.56) | 1.82 (0.70, 5.52) |
| New lesion on brain MRI | 3.75 (1.90, 7.41) | 3.69 (1.66, 8.22) | 3.67 (1.66, 8.09) | 3.42 (1.49, 9.00) |
| New GD+ lesion on brain MRI | 1.86 (0.77, 4.46) | 1.67 (0.61, 4.57) | 1.63 (0.58, 4.56) | 1.59 (0.50, 5.17) |
| New T2 lesion on brain MRI | 3.62 (1.80, 7.29) | 4.01 (1.75, 9.18) | 4.08 (1.80, 9.23) | 3.57 (1.52, 9.55) |
| Sustained disease progression | 1.27 (0.50, 3.25) | 1.21 (0.36, 4.01) | 1.01 (0.34, 3.02) | 0.89 (0.26, 2.43) |
| No relapse, new MRI lesion or sustained progression (NEDA) | 0.3 (0.17, 0.53) | 0.33 (0.17, 0.65) | 0.37 (0.20, 0.70) | 0.43 (0.19, 0.94) |

Legend: OR: Odds Ratio; CI: Confidence Interval; GD+: Gadolinium-enhancing; NEDA: No Evidence of Disease Activity. Estimated OR and 95% CI provided for each of the outcomes for each of the four approaches. OR>1 indicates higher probability of having an event on IFNb-1a compared to DMF.
