# Supplementary material for: Comparison of dimethyl fumarate and interferon outcomes in an MS cohort
Source: BMC Neurol. 2022 Jul 11;22:252. doi: 10.1186/s12883-022-02761-8 (PMC9277810; doi:10.1186/s12883-022-02761-8)
Supplement: Supplementary file 4 — Additional file 4: Supplementary table 4. Comparison of treatment groups among subjects who changed from previous treatment based on disease activity. [file 12883_2022_2761_MOESM4_ESM.docx]

Supplementary table 4: Comparison of treatment groups among subjects who changed from previous treatment based on disease activity

| Outcome | Unadjusted  OR (95%CI) | Regression adjustment for all confounding factors  OR (95%CI) | Regression adjustment for propensity score  OR (95%CI) | Inverse probability weighting  OR (95%CI) |
| --- | --- | --- | --- | --- |
| Clinical relapse(s) | 4.02 (1.15, 14.05) | 7.35 (1.36, 39.91) | 4.23 (1.02, 17.53) | 5.62 (1.08, 53.04) |
| New lesion on brain MRI | 2.92 (0.97, 8.82) | 3.19 (0.85, 11.99) | 3.57 (0.99, 12.83) | 3.53 (0.99, 22.57) |
| New GD+ lesion on brain MRI | 0.84 (0.22, 3.23) | 1.00 (0.21, 4.77) | 1.14 (0.24, 5.35) | 1.42 (0.09, 10.72) |
| New T2 lesion on brain MRI | 2.92 (0.97, 8.82) | 3.19 (0.85, 11.99) | 3.57 (0.99, 12.83) | 3.53 (0.99, 22.57) |
| Sustained disease progression | 1.31 (0.18, 9.80) | 2.07 (0.16, 27.18) | 1.37 (0.14, 13.69) | 1.26 (0, 100) |
| No relapse, new MRI lesion or sustained progression (NEDA) | 0.28 (0.11, 0.72) | 0.20 (0.06, 0.65) | 0.23 (0.07, 0.69) | 0.17 (0.05, 0.48) |

Legend: OR: Odds Ratio; CI: Confidence Interval; GD+: Gadolinium-enhancing; NEDA: No Evidence of Disease Activity. Estimated OR and 95% CI provided for each of the outcomes for each of the four approaches. OR>1 indicates higher probability of having an event on IFNb-1a compared to DMF.
