# Supplementary material for: Comparison of dimethyl fumarate and interferon outcomes in an MS cohort
Source: BMC Neurol. 2022 Jul 11;22:252. doi: 10.1186/s12883-022-02761-8 (PMC9277810; doi:10.1186/s12883-022-02761-8)
Supplement: Supplementary file 5 — Additional file 5: Supplementary table 5. Comparison of treatment groups among subjects who had a relapse in the previous year. [file 12883_2022_2761_MOESM5_ESM.docx]

Supplementary table 5: Comparison of treatment groups among subjects who had a relapse in the previous year

| Outcome | Unadjusted  OR (95%CI) | Regression adjustment for all confounding factors  OR (95%CI) | Regression adjustment for propensity score  OR (95%CI) | Inverse probability weighting  OR (95%CI) |
| --- | --- | --- | --- | --- |
| Clinical relapse(s) | 3.95 (1.37, 11.37) | 5.50 (1.59, 19.03) | 4.36 (1.41, 13.47) | 3.19 (0.91, 22.36) |
| New lesion on brain MRI | 3.23 (1.19, 8.74) | 3.72 (1.23, 11.24) | 3.49 (1.20, 10.14) | 3.31 (1.04, 16.71) |
| New GD+ lesion on brain MRI | 1.12 (0.36, 3.43) | 1.47 (0.43, 5.01) | 1.39 (0.42, 4.64) | 1.15 (0.26, 6.03) |
| New T2 lesion on brain MRI | 3.40 (1.17, 9.89) | 4.39 (1.33, 14.47) | 4.12 (1.31, 12.91) | 3.67 (1.13, 24.75) |
| Sustained disease progression | 1.30 (0.39, 4.35 | 0.88 (0.16, 4.69) | 0.86 (0.22, 3.32) | 0.99 (0.22, 5.18) |
| No relapse, new MRI lesion or sustained progression (NEDA) | 0.30 (0.14, 0.67) | 0.29 (0.12, 0.73) | 0.36 (0.15, 0.83) | 0.39 (0.12, 1.19) |

Legend: OR: Odds Ratio; CI: Confidence Interval; GD+: Gadolinium-enhancing; NEDA: No Evidence of Disease Activity. Estimated OR and 95% CI provided for each of the outcomes for each of the four approaches. OR>1 indicates higher probability of having an event on IFNb-1a compared to DMF.
