# Supplementary material for: Comparison of dimethyl fumarate and interferon outcomes in an MS cohort
Source: BMC Neurol. 2022 Jul 11;22:252. doi: 10.1186/s12883-022-02761-8 (PMC9277810; doi:10.1186/s12883-022-02761-8)
Supplement: Supplementary file 6 — Additional file 6: Supplementary table 6. Comparison of treatment groups among subjects who were between 18 and 55 at the time of treatment initiation. [file 12883_2022_2761_MOESM6_ESM.docx]

Supplementary table 6: Comparison of treatment groups among subjects who were between 18 and 55 at the time of treatment initiation

| Outcome | Unadjusted  OR (95%CI) | Regression adjustment for all confounding factors  OR (95%CI) | Regression adjustment for propensity score  OR (95%CI) | Inverse probability weighting  OR (95%CI) |
| --- | --- | --- | --- | --- |
| Clinical relapse(s) | 3.22 (1.63, 6.39) | 3.89 (1.65, 9.18) | 3.21 (1.45, 7.10) | 2.86 (1.00, 9.34) |
| New lesion on brain MRI | 4.28 (2.17, 8.45) | 5.01 (2.23, 11.29) | 4.91 (2.23, 10.85) | 4.55 (1.88, 11.84) |
| New GD+ lesion on brain MRI | 2.43 (1.01, 5.87) | 2.54 (0.92, 7.03) | 2.49 (0.90, 6.94) | 2.26 (0.71, 7.53) |
| New T2 lesion on brain MRI | 4.46 (2.19, 9.08) | 5.56 (2.40, 12.88) | 5.77 (2.52, 13.22) | 5.08 (2.06, 13.98) |
| Sustained disease progression | 1.51 (0.58, 3.92) | 1.32 (0.39, 4.45) | 1.15 (0.37, 3.51) | 0.94 (0.28, 2.64) |
| No relapse, new MRI lesion or sustained progression (NEDA) | 0.24 (0.13, 0.42) | 0.22 (0.11, 0.45) | 0.25 (0.13, 0.48) | 0.25 (0.10, 0.59) |

Legend: OR: Odds Ratio; CI: Confidence Interval; GD+: Gadolinium-enhancing; NEDA: No Evidence of Disease Activity. Estimated OR and 95% CI provided for each of the outcomes for each of the four approaches. OR>1 indicates higher probability of having an event on IFNb-1a compared to DMF.
