# Supplementary material for: Comparison of dimethyl fumarate and interferon outcomes in an MS cohort
Source: BMC Neurol. 2022 Jul 11;22:252. doi: 10.1186/s12883-022-02761-8 (PMC9277810; doi:10.1186/s12883-022-02761-8)
Supplement: Supplementary file 7 — Additional file 7: Supplementary table 7. Comparison of treatment groups among subjects who were between 18 and 55 at the time of treatment initiation and had a relapse in the previous year. [file 12883_2022_2761_MOESM7_ESM.docx]

Supplementary table 7: Comparison of treatment groups among subjects who were between 18 and 55 at the time of treatment initiation and had a relapse in the previous year

| Outcome | Unadjusted  OR (95%CI) | Regression adjustment for all confounding factors  OR (95%CI) | Regression adjustment for propensity score  OR (95%CI) | Inverse probability weighting  OR (95%CI) |
| --- | --- | --- | --- | --- |
| Clinical relapse(s) | 3.72 (1.28, 10.81) | 6.93 (1.76, 27.20) | 5.07 (1.55, 16.52) | 4.03 (1.10, 32.76) |
| New lesion on brain MRI | 3.03 (1.11, 8.29) | 4.48 (1.40, 14.37) | 4.26 (1.38, 13.12) | 4.04 (1.12, 22.57) |
| New GD+ lesion on brain MRI | 1.03 (0.33, 3.18) | 1.63 (0.47, 5.71) | 1.63 (0.47, 5.69) | 1.33 (0.27, 8.12) |
| New T2 lesion on brain MRI | 3.19 (1.09, 9.36) | 5.61 (1.56, 20.21) | 5.15 (1.53, 17.37) | 4.64 (1.20, 31.81) |
| Sustained disease progression | 1.20 (0.36, 4.07) | 0.82 (0.14, 4.80) | 0.92 (0.23, 3.65) | 1.00 (0.22, 5.71) |
| No relapse, new MRI lesion or sustained progression (NEDA) | 0.31 (0.14, 0.71) | 0.24 (0.09, 0.65) | 0.29 (0.12, 0.74) | 0.28 (0.08, 0.79) |

Legend: OR: Odds Ratio; CI: Confidence Interval; GD+: Gadolinium-enhancing; NEDA: No Evidence of Disease Activity. Estimated OR and 95% CI provided for each of the outcomes for each of the four approaches. OR>1 indicates higher probability of having an event on IFNb-1a compared to DMF.
